# Recent Advances in Nanomaterials for Pesticide Residue Detection: From Spectroscopic Analysis to Electrochemical Sensing

**DOI:** 10.3390/nano16130797

**Published:** 2026-06-27

**Authors:** Yue Niu, Mei Wang, Wei Lu, Bingliang Zhou, Xianghai Song, Quan Bu

**Affiliations:** 1 School of Agricultural Engineering, Jiangsu University, Zhenjiang 212013, China; 2222516096@stmail.ujs.edu.cn (Y.N.); 1000005405@ujs.edu.cn (W.L.); 1000004986@ujs.edu.cn (B.Z.); qbu@ujs.edu.cn (Q.B.); 2 Institute of the Green Chemistry and Chemical Technology, School of Chemistry and Chemical Engineering, Jiangsu University, Zhenjiang 212013, China; songxianghai@ujs.edu.cn

**Keywords:** nanomaterials, pesticide residue detection, spectroscopic analysis, electrochemical sensing, signal amplification

## Abstract

This review systematically summarizes the inherent characteristics and application superiorities of various nanomaterials, including metallic nanomaterials, metal oxides, carbon-based materials, metal–organic frameworks (MOFs), and quantum dots (QDs). State-of-the-art research progress is elaborated on the applications of these nanomaterials in multiple analytical techniques, such as surface-enhanced Raman spectroscopy (SERS), fluorescence spectroscopy, infrared spectroscopy, ultraviolet-visible spectroscopy, and electrochemistry. Furthermore, their pivotal functions in signal amplification, specific molecular recognition, and rapid analyte enrichment are thoroughly discussed. Additionally, this paper analyzes the prevailing challenges, including material heterogeneity, potential biosafety risks, poor anti-interference capacity against complex matrices, and the absence of unified industrial standardization. Future development directions are also proposed, involving green synthesis strategies, precise functional modification, portable intelligent detection, and simultaneous multi-component detection. This work aims to provide a reliable reference for further fundamental research and industrial translation of nanomaterials in the rapid and high-precision detection of pesticide residues.

## 1. Introduction

Pesticides are indispensable in modern agricultural production. They effectively control pests and pathogens to improve crop yield and quality, thereby securing the global food supply [1]. The extensive use of pesticides minimizes pest-induced crop losses, lowers agricultural costs, and increases farmers’ income [2]. Furthermore, pesticide application protects natural ecosystems from biological destruction [3]. However, pesticide residues pose severe threats to human health and the ecological environment [4]. Long-term intake of pesticide-contaminated food may cause immune impairment, endocrine disruption, neurological disorders, and even cancers [5]. Additionally, pesticide residues accumulate along food chains, exerting long-term adverse effects on wild organisms and ecosystems [6]. Therefore, it is critical to restrict pesticide residues in agricultural products within safe thresholds [7].

Pesticide residue detection is an essential guarantee for food safety and environmental protection. Efficient and accurate detection techniques enable timely identification and supervision of pesticide contaminants in agricultural commodities, preventing substandard products from entering the market and safeguarding consumer health [8]. Moreover, such detection strategies facilitate rational pesticide utilization and promote the sustainable development of modern agriculture.

Pesticides used in agricultural production cover a wide variety of categories, which differ significantly in physicochemical properties, action mechanisms, and residual characteristics. These inherent differences lead to distinct detection challenges and applicable analytical techniques, serving as the fundamental premise for pesticide residue detection research. The pesticides involved in this study and their corresponding categories are listed in Table 1.

Commonly adopted conventional detection techniques include gas chromatography (GC), high-performance liquid chromatography (HPLC), GC-mass spectrometry (GC-MS), liquid chromatography-MS (LC-MS), HPLC-MS [9], and enzyme inhibition assays [10,11]. GC delivers high sensitivity at the ppb level and satisfactory separation performance, yet it is incompatible with polar pesticides and requires expensive instrumentation. HPLC is suitable for polar pesticide detection but suffers from long analytical durations and excessive organic solvent consumption. Hyphenated mass spectrometry techniques combine chromatographic separation with mass spectrometric identification, achieving limits of detection (LODs) below 0.1 ppb; nevertheless, their high maintenance costs and operational complexity require professional technicians. Enzyme inhibition methods allow rapid screening but are only applicable to organophosphorus and carbamate pesticides, with an inherent false-positive rate. In addition, thin-layer chromatography features simple operation but low sensitivity, whereas immunoassays exhibit high specificity but are limited by complicated antibody preparation and import dependence. Traditional methods are universally constrained by cumbersome pretreatment and destructive sample processing, which cannot meet the requirements of real-time on-site detection in modern agriculture [12]. Hence, novel detection technologies are urgently required to overcome these drawbacks for improved detection efficiency and reduced costs.

Emerging pesticide detection technologies integrate nanotechnology, spectroscopy, and biosensing to enhance analytical performance [13]. For example, surface-enhanced Raman scattering (SERS) achieves single-molecule detection sensitivity based on the localized electromagnetic field effect of nanostructured substrates [14,15]. Hyperspectral imaging combined with the ResNet algorithm acquires spatial distribution information of pesticides, overcoming the limitation of discrete data obtained by chromatographic techniques. Molecularly imprinted sensors achieve a specific recognition rate of 98.6% for organophosphorus pesticides, effectively reducing the 15–30% cross-reaction rate of conventional enzyme inhibition methods [16]. In addition, magnetic molecularly imprinted polymers (MMIPs) supported by nano-sized ferroferric oxide and other nanomaterials feature specific molecular recognition and rapid magnetic separation, enabling efficient extraction of various pesticides with strong anti-matrix interference, excellent adsorption capacity and favorable reusability [17]. Most of these innovative detection techniques stem from advances in nanotechnology [18]. Accordingly, nanotechnology has attracted extensive research interest for developing novel pesticide detection approaches [19].

As fundamental components of nanotechnology, nanomaterials exhibit revolutionary application potential in pesticide detection with prominent superiority in sensitivity, selectivity, and detection efficiency [20]. In terms of detection sensitivity, the localized surface plasmon resonance (LSPR) of noble metal nanoparticles enables SERS to achieve a limit of detection (LOD) down to 0.01 μg/kg, three orders of magnitude lower than conventional detection methods [21]. Combined with spectral preprocessing and partial least squares (PLS) regression modeling, SERS further reduces detection limits and yields satisfactory recoveries [22]. Enabled by nanomaterials for distinctive Raman fingerprint acquisition, SERS coupled with transfer-learning-based 1D- convolutional neural network (CNN) improves the identification accuracy of eight pesticides and cuts sample demand for rapid on-site testing [23]. In terms of selectivity, graphene oxide (GO) captures organophosphorus pesticides via π-π stacking interaction, decreasing the cross-reaction rate from 30% (enzyme-linked immunosorbent assay) to below 2%. Molecularly imprinted nanopolymers achieve a recognition accuracy of 98.6% for neonicotinoid pesticides, outperforming the 85% accuracy of conventional biosensors [24]. For detection efficiency, self-cleaning titanium dioxide (TiO_2_)@Ag nanosubstrates support 50 reuse cycles, reducing the single-test cost to 1/20 of that of traditional approaches. The integration of nanocomposites with QuEChERS (Quick, Easy, Cheap, Effective, Rugged, Safe) pretreatment achieves a purification efficiency of 85–95% for complex matrices (e.g., 2,4-D in milk), solving the unstable recovery issue of traditional solid-phase extraction [25]. A synergistic nanoparticle/SERS/molecularly imprinted polymer (MIP) system employs Au/Ag nanoparticles for electromagnetic enhancement and MIPs for specific pesticide recognition and enrichment. This system produces sensitive SERS responses toward the P=O/P=S characteristic groups of organophosphorus pesticides, achieving ultra-low LODs at nM/ng/kg levels with wide linear ranges and excellent reproducibility. It effectively suppresses matrix interference and simplifies pretreatment procedures [26,27]. Similarly, the combination of MIPs and QuEChERS eliminates matrix contaminants and enables high-selectivity detection in complex samples [28]. Precipitation polymerization further facilitates scalable material preparation and expands detection scenarios from laboratory analysis to field testing. Owing to their multifunctional characteristics, nanomaterials are promoting the intelligent (hyperspectral imaging), miniaturized (microfluidic chips), and automated (nanorobot sampling) development of pesticide detection.

This review systematically summarizes the research progress of nanomaterials in pesticide residue detection. The classification and intrinsic properties of diverse nanomaterials are introduced, and their applications in spectroscopic, electrochemical, and biological detection techniques are discussed, alongside practical detection cases in different complex matrices. The current limitations and challenges of nanomaterial-based detection are analyzed, and future development directions are prospected. This paper concludes the research achievements and scientific significance of related studies, aiming to provide reliable references for subsequent fundamental research and industrialized application in this field.

## 2. Types and Characteristics of Nanomaterials for Pesticide Detection

### 2.1. Metal Nanomaterials

**Figure 1:**
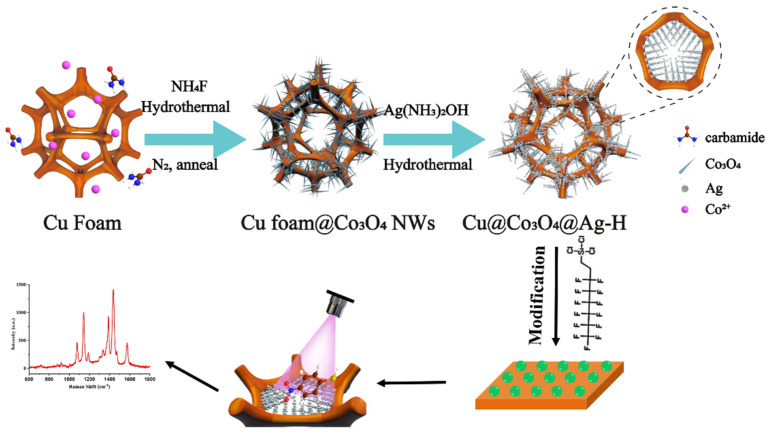
Ag NPs-based Cu@Co_3_O_4_@Ag Array SERS Sensor for Rapid Trace Droplet Detection in Fruits and Vegetables [31].

Common metallic nanomaterials for pesticide detection mainly include gold nanoparticles (AuNPs), silver nanoparticles (AgNPs), and platinum nanoparticles [29,30]. Silver-based materials can be fabricated into multi-level functional substrates. For example, a hierarchical Co_3_O_4_/Ag nano-SERS substrate is constructed with copper foam as the support; relying on silver plasmon enhancement, it achieves ultrasensitive detection of thiram, and hydrophobic modification enables rapid sampling of tiny droplets for on-site pesticide screening by wiping fruit and vegetable surfaces [31] (Figure 1). Such nanomaterials possess unique physicochemical properties and have made substantial research advances in pesticide detection [32,33,34]. Their prominent characteristics involve surface plasmon resonance (SPR) effect, favorable electrical conductivity, superior catalytic activity, and good biocompatibility. Among these properties, the SPR effect serves as the core mechanism for high-sensitivity detection by significantly amplifying the detection signals of pesticide molecules [35]. The favorable conductivity and catalytic activity accelerate electron transfer during detection processes to improve response speed, while excellent biocompatibility ensures their stable applicability in biological detection systems [36,37]. Metallic nanomaterials exhibit distinct advantages over conventional detection materials. They enable trace and ultra-trace pesticide detection with high sensitivity and rapid response. Moreover, certain metallic nanomaterials support visual detection without sophisticated instruments, facilitating convenient on-site screening [38]. Current research priorities focus on morphology regulation and surface modification of metallic nanomaterials. The optimization of fabrication procedures can regulate the size and morphology of nanoparticles, and surface modification strategies improve material stability and specificity while mitigating nanoparticle aggregation [39,40]. Furthermore, diversified detection systems based on metallic nanomaterials, including SERS platforms, electrochemical sensors, and immunoassays, have been constructed for the detection of organophosphorus, pyrethroid, and carbamate pesticides. These systems continuously optimize detection sensitivity and specificity to broaden detection ranges [41,42]. To date, metallic nanomaterials have been successfully applied for pesticide residue analysis in various matrices such as vegetables, fruits, and water, providing reliable technical support for rapid and precise pesticide detection. Nevertheless, challenges including high large-scale preparation costs and insufficient stability still need to be addressed to promote their industrialization.

### 2.2. Metal Oxide Nanomaterials

Metal oxide nanomaterials are widely adopted for pesticide detection, mainly including TiO_2_, zinc oxide (ZnO), tin dioxide (SnO_2_), iron oxide (Fe_3_O_4_), and copper oxide (CuO). Benefiting from unique physicochemical performances, metal oxide nanomaterials occupy an essential position in pesticide detection with remarkable application potential, and considerable research progress has been achieved in recent years [43,44]. Their inherent characteristics contain high specific surface area, strong adsorption capacity, excellent photocatalytic performance, and favorable electrochemical activity [45,46]. The high specific surface area provides abundant active sites for the efficient adsorption and enrichment of pesticide molecules, which is particularly suitable for trace pesticide analysis. Strong adsorption capability enhances the specific binding affinity toward pesticide molecules and reduces matrix interference. The excellent photocatalytic performance generates active free radicals under illumination, thereby realizing pesticide degradation and signal amplification to improve detection sensitivity. Moreover, favorable electrochemical activity accelerates interfacial electron transfer and optimizes detection response efficiency.

Compared with traditional detection materials, metal oxide nanomaterials possess stronger adsorption capacity for pesticide molecules, enabling effective enrichment of trace pesticides and achieving detection levels down to nanogram or even picogram scales. These nanomaterials exhibit superior detection stability and strong anti-interference capability, which makes them applicable for complex matrices including soil, agricultural products, and water. Additionally, they feature facile fabrication procedures and lower production costs than metallic nanomaterials, facilitating large-scale manufacturing. Their excellent compatibility enables integration with spectroscopic, electrochemical, and biological technologies to construct multifunctional detection platforms for rapid and precise pesticide monitoring.

Current research mainly focuses on two major directions: material modification and detection system construction. On the one hand, surface modification, morphology regulation, and composite modification strategies are employed to optimize the adsorption, photocatalytic, and electrochemical properties of metal oxide nanomaterials. These approaches mitigate particle aggregation and improve detection specificity and stability; for instance, noble metal doping and carbon-based composite modification can further strengthen signal amplification capability. On the other hand, diverse detection systems based on metal oxide nanomaterials, such as spectroscopic platforms, electrochemical sensors, and photocatalytic detection systems, have been developed for organophosphorus, pyrethroid, and carbamate pesticides. These systems have been extensively applied for trace pesticide detection in agricultural products, water, and soil matrices. Despite the encouraging achievements, several limitations remain, such as insufficient detection specificity and unsatisfactory anti-interference performance in complex matrices. Future research will prioritize the exploration of innovative metal oxide nanomaterials, the development of low-cost large-scale preparation techniques, and the miniaturization and intelligent upgrading of detection systems to promote their industrialization in pesticide detection.

### 2.3. Carbon-Based Nanomaterials

With unique structural characteristics and excellent intrinsic performances, carbon-based nanomaterials have emerged as core candidates for the rapid and highly sensitive detection of pesticide residues, which are widely applied in food safety and environmental monitoring. Such materials possess low toxicity and high fluorescence quantum yield. Combined with recognition units such as enzymes and aptamers, carbon-based nanomaterials can construct high-sensitivity sensing platforms with detection limits down to the pg/mL level, providing an innovative technical route for the rapid trace analysis of pesticides in food and environmental samples [47]. According to dimensional classification, common carbon-based nanomaterials include zero-dimensional fullerenes and carbon quantum dots (CQDs), one-dimensional carbon nanotubes, two-dimensional graphene and reduced graphene oxide (rGO), as well as three-dimensional porous carbon materials. In terms of inherent properties, carbon-based nanomaterials generally exhibit ultra-high specific surface area, excellent electrical and thermal conductivity, favorable chemical stability, and facile functionalization capability. These characteristics enable them to adapt to the identification requirements of diverse pesticide molecules through surface modification [48]. Carbon-based nanomaterials present prominent advantages in pesticide detection. Firstly, they possess extremely high sensitivity: carbon nanotubes and graphene accelerate electron transfer, while Carbon-based nanomaterials such as CQDs realize nanogram- and picogram-level residue detection relying on strong fluorescence properties, achieving much lower detection limits than conventional methods [49]. Secondly, they provide satisfactory selectivity. The integration of molecular imprinting and enzyme immobilization technologies enables the precise identification of target pesticides and mitigates matrix interference [50]. Thirdly, these materials achieve rapid detection responses without cumbersome pretreatment, making them suitable for on-site screening. Fourthly, they have low fabrication costs and excellent stability, exhibiting better environmental tolerance and reusability than biological enzymes. In terms of research progress, modified electrodes incorporated with multi-walled carbon nanotubes have realized the ultrasensitive detection of organophosphorus and carbamate pesticides with detection limits as low as ng/L in electrochemical sensing systems. For fluorescence sensing, nitrogen/sulfur-doped CQDs achieve visual quantitative detection of glyphosate and parathion based on the fluorescence quenching and recovery mechanism [51]. Colorimetric sensing realizes rapid naked-eye discrimination relying on the peroxidase-like activity of carbon-based nanozymes. Current research is advancing toward multifunctional integration, portability, and intelligence. For example, the combination of carbon-based nanomaterials with molecular imprinting and microfluidic chips facilitates the development of on-site rapid detection devices. Nevertheless, several challenges remain, including batch homogeneity during large-scale preparation, anti-interference capacity in complex matrices, and long-term stability. In the future, green synthesis, precise functional modification, and multi-technology integration will further promote the practicalization and industrialization of carbon-based nanomaterials in pesticide residue detection.

### 2.4. Other Novel Nanomaterials

Novel nanomaterials, such as quantum dots (QDs), metal–organic frameworks (MOFs), and various nanocomposites, have demonstrated tremendous application potential in pesticide residue detection due to their structural controllability and excellent optical and electrical properties [52,53]. QDs possess high fluorescence quantum yields, tunable emission wavelengths, and favorable photobleaching resistance. MOFs are characterized by ultra-large specific surface areas, adjustable pore structures, and facile functional modification. Moreover, nanocomposites integrate the merits of diverse components to realize signal amplification and synergistic recognition [54]. These nanomaterials exhibit prominent advantages in pesticide detection. QDs can fabricate highly sensitive fluorescence sensing systems to achieve rapid responses toward trace pesticides [55]. MOFs can act as enrichment carriers and recognition units to substantially improve detection selectivity and sensitivity, while nanocomposites effectively optimize interfacial mass transfer efficiency and lower LODs. In current research, fluorescence sensors based on CdTe QDs and carbon dots have achieved the highly sensitive detection of paraquat, organophosphorus, and pyrethroid pesticides [56]. MOF-modified electrodes and fluorescent probes exhibit superior performance in the simultaneous detection of multiple pesticides. In addition, diverse functional nanocomposites combined with electrochemical, colorimetric, and Raman techniques greatly enhance the detection accuracy of pesticides in complex matrices. Current research is evolving toward in situ, real-time, and portable detection, and several sensing systems are applicable for practical sample analysis. Nevertheless, several challenges remain, including poor synthesis controllability, biosafety risks, and insufficient anti-interference capability in real samples. In the future, green synthesis and precise functional modification will further promote the practical implementation of these nanomaterials in on-site rapid pesticide detection.

## 3. Main Application Technologies and Research Progress of Nanomaterials in Pesticide Detection

Benefiting from excellent optical properties, favorable electrical conductivity, and high specific surface areas, nanomaterials have been regarded as ideal functional materials for the efficient detection of pesticide residues. Nanomaterial-based pesticide detection technologies have developed rapidly with continuously optimized application systems. SERS technology is widely utilized for the rapid screening of trace pesticides owing to its unique signal amplification capability. Fluorescence spectroscopy enables sensitive and visualized pesticide analysis due to its simple operation and rapid response. Moreover, other spectroscopic techniques, including ultraviolet and infrared spectroscopy, have been progressively optimized and upgraded. In parallel, innovative detection methods based on the superior electrochemical performances of nanomaterials continuously emerge, compensating for the limitations of individual detection technologies. The coordinated development of diverse analytical techniques provides novel insights and technical support for the precise detection of pesticide residues in agricultural products.

### 3.1. SERS Detection Technology

**Figure 2:**
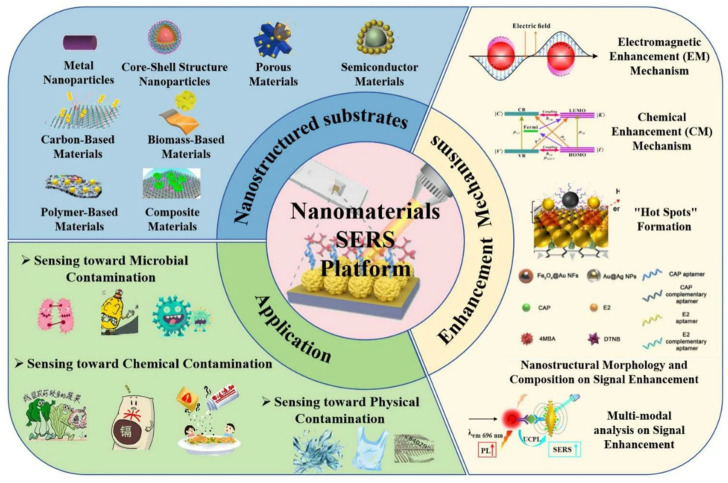
Nanomaterial-Based SERS Platform: Substrates, Enhancement Mechanisms, and Applications [60].

Conventional Raman spectroscopy is plagued by low detection sensitivity and narrow detection range. As a powerful alternative, SERS overcomes these drawbacks and breaks the application bottleneck of conventional spectroscopic detection, laying a crucial technical foundation for the precise trace-level identification and quantification of various microscale analytes [57,58]. SERS commonly adopts noble metal nanomaterials such as gold and silver as functional substrates. Benefiting from the intrinsic SPR effect of these substrates, the Raman scattering signals of target molecules can be amplified by millions of times to greatly improve detection sensitivity [59]. Nanomaterials constitute the core for ultrasensitive SERS detection, and the overall signal enhancement stems from the synergistic effect of electromagnetic enhancement and chemical enhancement, which optimizes the Raman spectral response performance effectively (Figure 2) [60].

Between the two enhancement mechanisms, electromagnetic enhancement dominates signal amplification in SERS systems. Upon optical excitation, noble metal nanostructures produce LSPR and generate intense electric field hotspots at nanogaps and sharp tips of nanoparticles. The local electric field can be enhanced by 10^2^–10^7^ orders of magnitude, enabling efficient amplification of Raman signals from target molecules [61]. In contrast, chemical enhancement serves as an auxiliary enhancement route. Relying on interfacial charge transfer between nanosubstrates and target molecules, this mechanism alters molecular polarizability and Raman scattering cross-section to achieve mild signal amplification with enhancement factors ranging from 10 to 10^2^, and such effect is more pronounced in SERS systems constructed with semiconductors and two-dimensional materials [62,63].

From the perspective of interfacial molecular interaction, SERS enhancement efficiency is closely correlated with substrate surface selection rules and spatial conformation of adsorbed analytes, and strongly relies on reactive functional groups including thiol, amino, organophosphorus P=S and P=O groups that can form specific chemical adsorption with gold and silver substrates. The chemical structure of pesticide molecules directly determines their binding affinity toward noble metal nano-substrates: pesticides carrying the aforementioned active groups can directly adsorb onto bare noble metal surfaces and obtain drastically enhanced Raman signals via preformed electromagnetic hotspots, hence realizing quantitative detection without extra substrate modification. Nevertheless, most hydrophilic and high-polarity pesticides lack these anchoring functional groups and fail to immobilize stably on pristine Au/Ag substrates. During detection, impurities from food matrices compete for available adsorption sites, resulting in aggravated baseline noise and weakened signal intensity and eventually hindering accurate quantification [60]. To solve this problem, MIPs are utilized for substrate functionalization. Combined with the size-sieving effect of imprinted cavities and specific molecular recognition interactions such as hydrogen bonding and hydrophobic interaction, MIP modifiers can selectively capture target pesticides and remedy the weak adsorption of bare noble metals toward such analytes, so as to ensure favorable accuracy and stability of detection.

In recent studies, nanomaterials have been evolving toward compositional hybridization and functional modification. Precious metal-based nanomaterials (e.g., nanostars, nanorods, and core–shell structures) enable the precise modulation of LSPR, achieving enhancement factors ranging from 10^9^ to 10^12^, which approaches the single-molecule detection level [41,64]. For instance, Zhu et al. constructed a rapid SERS detection platform utilizing gold nanostars (Au NSs) as precious metal-enhanced substrates for the trace quantitative analysis of chlorpyrifos residues in tea. Au NSs fabricated via the seed-mediated growth method possess abundant high-density hot spots on their surfaces, yielding a Raman enhancement factor of 1.06 × 10^5^ and efficiently amplifying the characteristic signals of pesticide molecules. After spectral pretreatment including baseline correction, scattering correction, and smooth normalization, PLS combined with four intelligent variable selection algorithms (MCUVE, CARS, IRIV, and VISSA) were employed for model establishment. The VISSA model exhibited the optimal prediction performance with a determination coefficient (R^2^) of 0.9892 and a residual predictive deviation (RPD) of 9.6246 in the prediction set. The LOD was as low as 3.9 × 10^−4^ μg/g, which was considerably lower than the maximum residue limit stipulated by the European Union. This method possessed excellent repeatability and reproducibility, and no significant difference was observed compared with GC-MS. The results fully demonstrate the high sensitivity, rapidity, and non-destructive superiority of precious metal-based SERS techniques, providing an accurate and efficient alternative for the on-site rapid detection of pesticide residues in tea [64].

Semiconductors and two-dimensional materials can improve SERS activity through doping and defect engineering, effectively addressing the high cost and poor stability limitations of precious metals [65,66]. To overcome the inherent drawbacks of conventional Au and Ag substrates, such as high fabrication costs and inferior chemical stability, Gao et al. focused on semiconductors and two-dimensional materials. High-density hot spots were precisely fabricated via doping regulation and defect engineering strategies, which significantly improved Raman enhancement efficiency and detection sensitivity. The proposed substrates realized the highly selective trace identification of typical heavy metals, including mercury, arsenic, cadmium, lead, and chromium. Such innovative substrates possess the advantages of low cost, environmental friendliness, excellent environmental stability, and favorable signal reproducibility. Combined with chemometric analysis and density functional theory calculation, these substrates effectively break the application restrictions of traditional SERS techniques, offering a sustainable and efficient technical route for the rapid, accurate, and large-scale on-site detection of heavy metals in food [67]. Furthermore, Guo et al. comprehensively summarized the fundamental principles of optical sensing systems based on fluorescence, SERS, SPR, and colorimetric methods. The enhancement capability of metal, semiconductor, and carbon-based nanomaterials for optical sensors was systematically reviewed for the detection of pesticides, heavy metals, biotoxins, and foodborne pathogens. Additionally, the limitations and future prospects of optical sensors were discussed, highlighting three key research findings: (a) artificial intelligence-assisted nanomaterial design; (b) smartphone-integrated portable detection systems; (c) self-validating dual-mode sensors. This study aims to promote the strategic advancement of high-precision sensing technologies and ultimately ensure food safety [59].

**Figure 3:**
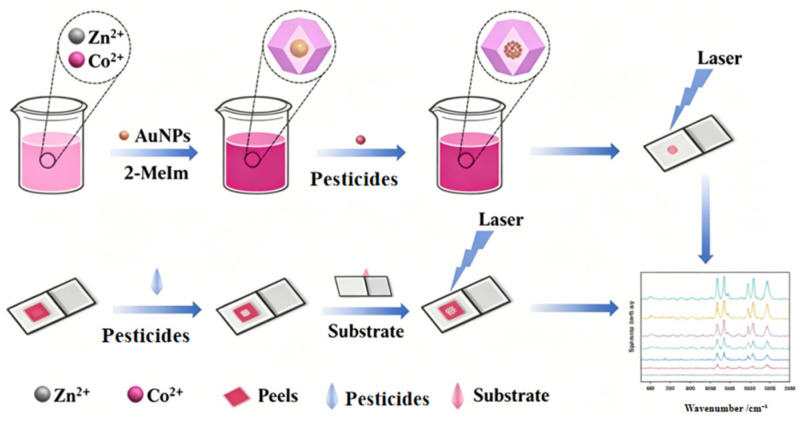
A core–shell structured AuNPs@ZnCo-MOF SERS substrate for sensitive and selective detection of thiram [68].

Composite SERS substrates fabricated by integrating MOFs and porous carbon-supported metal nanoparticles possess efficient molecular enrichment capability, remarkable Raman enhancement performance, and excellent molecular selectivity, which are suitable for the precise detection of trace components in complex matrices. For instance, Fu et al. synthesized core–shell structured AuNPs@ZnCo-MOF composite SERS-active substrates for the highly sensitive and selective quantitative detection of ziram. Owing to its high specific surface area, ordered porous structure, and strong adsorption capacity, the bimetallic ZnCo-MOF shell can efficiently enrich target molecules. Meanwhile, the shell stabilizes AuNPs, inhibits nanoparticle aggregation, and resists external environmental interference, enabling the substrate to remain stable for 28 days at room temperature. The optimized composite substrate achieved a high SERS enhancement factor of 3.51 × 10^6^, with a low LOD of 1 × 10^−7^ mol/L for ziram. The linear range was determined to be 1 × 10^−7^ to 1 × 10^−4^ mol/L with a correlation coefficient (R^2^) of 0.9969. In practical apple and tomato peel samples, the calculated spike recovery ranged from 83.45% to 99.61%, while the relative standard deviation (RSD) was lower than 8%. Benefiting from the size-sieving effect of MOFs and the specific Au-S interaction, the prepared substrate exhibited outstanding anti-interference ability and molecular selectivity. This work effectively combines the porous enrichment advantages of MOFs with the electromagnetic enhancement mechanism of precious metal nanoparticles, providing an efficient and reliable strategy for the on-site rapid SERS detection of trace pesticide residues in fruits and vegetables (Figure 3) [68].

**Figure 4:**
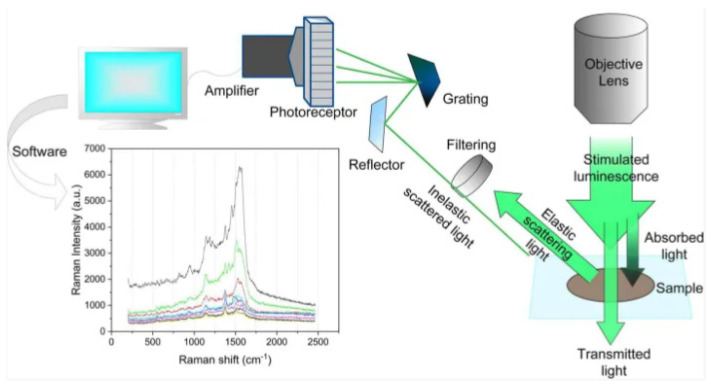
IOT environment for detection of thiram pesticide residues on fruit surface based on AuNPs SERS and KPCA-PLS algorithm [14].

The modification of SERS substrates with biomolecules such as aptamers and antibodies can endow SERS technology with specific recognition capability toward target analytes, greatly promoting its practical application in on-site rapid detection [6,69]. Cai et al. fabricated core–shell Au@Ag nanoparticles via a seed-mediated growth method as SERS-active substrates. Combined with a portable Raman spectrometer, a label-free sensing system was constructed for the simultaneous detection of acetamiprid and thiram pesticide residues. The strongest Raman signal enhancement was obtained by optimizing the thickness of the silver shell, enabling the precise quantitative analysis of the two pesticides in apple juice and orange juice matrices. The LODs for acetamiprid and thiram in apple juice were 1.22 μM and 0.076 μM, respectively. The spike recoveries ranged from 90.2% to 122.12%. This method features non-interfering characteristic peaks and simple detection procedures, and its detection sensitivity satisfies the maximum residue limits stipulated by the Environmental Protection Agency. This study provides an efficient SERS strategy for the simultaneous on-site detection of multiple pesticide residues in fruits and vegetables [37] (Figure 4).

The continuous innovation of nanomaterials provides essential technical support for the iterative upgrading of SERS detection technology, facilitating its extensive applications in environmental monitoring, food safety, and other fields. Particularly in trace analysis, SERS exhibits irreplaceable technical value and broad application prospects owing to its high sensitivity and rapid response [70]. In the field of pesticide residue detection, SERS sensing technology based on metal nanocomposite substrates has been continuously optimized, forming multiple mainstream research directions. Firstly, Au@Ag core–shell bimetallic enhancement systems are fabricated to integrate the excellent stability of gold and the strong electromagnetic enhancement effect of silver. Such structures overcome the trade-off between sensitivity and stability of single-metal substrates and realize the ultra-sensitive trace detection of various pesticides including acetamiprid, thiram, and thiabendazole in juice samples [37]. Secondly, flexible SERS substrates based on poly (dimethyl diallyl ammonium chloride)/Poly (sodium 4-styrenesulfonate) (PDADMAC/PSS)-modified filter paper are fabricated by the ordered assembly of Au@Ag nanorods on cellulose surfaces. The bendable and attachable flexible sensing interface enables the in situ, non-destructive, and rapid screening of non-systemic pesticides on the surfaces of fruits and vegetables such as apples and tomatoes, proposing a novel “attachment-detection” analytical mode [71]. Thirdly, the combination of immuno-recognition and SERS technology significantly improves the anti-interference capability and targeting accuracy relying on the specific binding ability of antibodies, achieving the simultaneous quantitative determination of acetamiprid and carbendazim in complex matrices [72]. Fourthly, dual-modal detection methods integrating naked-eye semi-quantitative observation and precise SERS quantification are established. This strategy balances rapid on-site screening and high-accuracy laboratory measurement, greatly improving the practicality and field applicability of sensing platforms [73]. Fifthly, multifunctional ZnO/Ag/MIPs composite substrates with molecular imprinting and photocatalytic self-cleaning performance are developed. The optimized substrates simultaneously enhance detection sensitivity, molecular selectivity, and ultraviolet regeneration capacity, effectively solving the poor selectivity and low reusability limitations of conventional SERS substrates [74].

Numerous studies have verified that molecularly imprinted SERS sensing platforms modified with metal nanoparticles possess excellent detection sensitivity and anti-interference recognition performance toward hydrophilic and strongly polar pesticides in complex food matrices, emerging as a popular research direction in this field. Typical pesticides including 2,4-D, triazine herbicides and neonicotinoid pesticides have no sulfhydryl groups or other highly active functional groups for specific adsorption on Au/Ag SERS substrates. These highly polar and hydrophilic pesticide analytes are prone to interference from food matrix components, making them typical detection objects suitable for MIP-functionalized SERS substrates [75]. She et al. constructed a sensing system by integrating MIPs with specific recognition capability for 2,4-D onto silver nanoparticle-modified SERS substrates, which enabled the precise separation and detection of 2,4-D in milk. This sensing system achieved an ultra-low LOD of 0.006 ppm at the characteristic peak of 391 cm^−1^, with a linear range covering international statutory limits. Combined with optimized QuEChERS pretreatment, the spike recoveries ranged from 85% to 95%, and the entire detection procedure was completed within 20 min, proving its feasibility for the rapid screening of dairy products [25]. To meet the detection requirements for hydrophilic and polar pesticides, Cao et al. optimized functional monomers and substrate architectures to improve the performance of SERS-MIPs sensors. An AuNP-MIP composite platform was fabricated for the simultaneous detection of prometryn and simetryn in rice and wheat. Sodium chloride-induced nanoparticle aggregation was employed to generate abundant electromagnetic hot spots for signal amplification, effectively eliminating complex matrix interference from grain samples. This method exhibited a linear range of 0.02–0.5 μg/mL and an LOD of 20 μg/kg with recoveries of 72.7–90.9%, providing a reliable analytical strategy for the multi-residue detection of triazine herbicides [28]. Additionally, magnetic AuNP-MIP composite SERS substrates were successfully applied for the rapid detection of acetamiprid and thiacloprid in peaches and pears. Benefiting from the well-designed core–shell structure, the sensing system realized ultrafast analyte enrichment and magnetic separation within 1 min. The fabricated sensor displayed a linear range of 1–20 μg/g, low LODs of 23.7–68.8 ng/g, and acceptable recoveries of 73.5–112.8%. Moreover, the composite materials could be reused 5–6 times, offering an efficient strategy for the trace detection of neonicotinoid pesticide residues [76].

Apart from molecularly imprinted composite systems, the combination of label-free flexible SERS sensing based on metal nanocomposite substrates and intelligent algorithms further expands the application scenarios and detection performance boundaries for pesticide residue analysis. For example, Tang et al. fabricated SERS substrates using AuNPs as enhancement materials and combined kernel principal component analysis (KPCA) with PLS algorithms to detect thiram residues on fruit surfaces. A nonlinear feature extraction model was constructed by optimizing spectral pretreatment procedures and kernel function parameters, which effectively eliminated fluorescence background and noise interference and realized the accurate identification of trace pesticides. The results demonstrated that the developed SERS sensor achieved a low LOD of 0.1 μg/g for thiram, exhibiting superior detection accuracy and stability compared with conventional principal component analysis (PCA). This method provides a reliable technical scheme for the on-site, rapid, and ultrasensitive detection of pesticide residues on fruit surfaces (Figure 4) [14]. Furthermore, Chen et al. fabricated label-free SERS substrates using gold nanorods and integrated chemometric algorithms for the rapid quantitative detection of thiabendazole in citrus fruits. After spectral pretreatment and PCA dimensionality reduction, the support vector machine classification accuracy reached 99.1667%, while the genetic algorithm-PLS (GA-PLS) model achieved an ultra-low LOD of 0.33 μg/mL. The established method was efficient and reliable, and its detection results were consistent with those obtained by HPLC [77]. Moreover, Guo et al. synthesized a flexible PAN/Cu_2_O@Ag/Au@Ag nanocomposite SERS substrate with integrated Raman enhancement and photocatalytic self-cleaning functions for cyclic utilization. Coupled with a deep learning CNN algorithm, the substrate enabled the in situ and non-destructive detection of thiram on apple surfaces, with a high correlation coefficient of 0.9963 and a low LOD of 0.020 mg/L. The above studies provide advanced technical insights for the high-sensitivity and rapid on-site detection of pesticide residues in fruits and vegetables [78].

Currently, SERS detection technology is evolving toward portability, multi-component simultaneous analysis, and in situ real-time monitoring [79,80]. The primary challenges lie in substrate homogeneity, anti-interference capability in complex matrices, and long-term stability. In the future, the integration of green synthesis strategies for nanomaterials and intelligent algorithms will further accelerate the industrialization process of SERS technology for practical pesticide detection [81].

### 3.2. Fluorescence Spectroscopy Detection Technology

Fluorescence spectroscopy has emerged as a mainstream technique for on-site screening and quantitative analysis of pesticide residues due to its rapid response, high sensitivity, simple operation and non-destructive sampling [82]. Traditional fluorescent sensors suffer from inherent drawbacks including light source fluctuation, matrix background interference, spectral overlap of multiple components, poor selectivity and limited performance in trace detection, which restrict their high-throughput on-site applications [83,84]. The development of novel nanomaterials provides crucial support for the performance optimization of fluorescence sensing systems [85,86].

In recent years, various advanced functional nanomaterials such as MOFs, nanozymes, upconversion nanoparticles (UCNPs) and carbon-based nanomaterials have been extensively explored. Relying on three classic effects, namely fluorescence resonance energy transfer (FRET), aggregation-induced emission (AIE) and LSPR, these materials comprehensively improve the sensitivity, selectivity and anti-interference capability of fluorescence sensors, serving as core strategies to address the limitations of conventional technologies [87,88]. Specifically, FRET enables the regulation of “on-off” fluorescence signals and lays a foundation for constructing highly sensitive sensing interfaces [89]. The AIE effect eliminates the aggregation-caused quenching effect of conventional organic fluorescent molecules. Meanwhile, LSPR derived from metallic nanoparticles amplifies optical signals via metal-enhanced fluorescence [90]. Collectively, these three effects facilitate the fabrication of high-performance fluorescence detection systems.

Currently, nanomaterial-based fluorescence detection systems have been developed into multiple mature application directions. Magnetic molecularly imprinted fluorescent composites integrate enrichment and detection functions. Using SiO_2_ and Fe_3_O_4_ as carriers and carbon dots as fluorescent signal units, these composites realize rapid identification of organophosphorus pesticides with a total detection time of only 2 min, and exhibit favorable selectivity and reusability in cucumber samples [91]. MOF-based fluorescent visual sensors possess large specific surface areas and abundant active sites, thus featuring fast mass transfer and strong anti-interference ability. The LOD for methyl parathion reaches as low as 4.95 μg/L, demonstrating great potential for the development of portable on-site detection devices [92]. To resolve the problem of overlapping spectra in multi-pesticide analysis, the combination of fluorescent nanomaterials with chemometrics achieves spectral decoupling and simultaneous quantitative detection of multiple pesticides in water samples, with the coefficient of determination ranging from 0.9847 to 0.9942 [93].

Nanozyme-mediated dual-mode fluorescence sensing has become a hot research topic in recent years. Nanozymes integrate the intrinsic properties of nanomaterials and enzyme-like catalytic activity, and can modulate optical signals through catalytic reactions. The dual-mode detection strategy further enhances the reliability and environmental adaptability of analytical methods. Li et al. constructed a fluorescent-colorimetric dual-mode sensing platform for glyphosate detection using CeO_2_ nanozymes combined with DNAzymes and click chemistry. Benefiting from the reversible Ce^3+^/Ce^4+^ redox cycle, stable catalytic performance was achieved. Both detection modes obtained microgram-level LODs, and satisfactory recovery rates and anti-interference performance were verified in tap water and soybean samples [94]. Furthermore, ratiometric fluorescence and functional modification with aptamers or MIPs have become prevailing strategies to reduce systematic errors and improve anti-interference capacity. Dual-emission ratiometric fluorescent probes fabricated from carbon-silica nanodots and gold nanoclusters can effectively counteract signal fluctuations caused by environmental factors and instrumental errors. Low-toxic carbon nanomaterials including CQDs and graphene QDs possess tunable luminescence and excellent biocompatibility, and have been widely applied in the trace screening of various pesticides, with some systems achieving LODs down to the pg/mL level [43,95].

Rare earth-doped UCNPs are excited by near-infrared light, which effectively avoids interference from sample autofluorescence and scattered light. Sensors based on UCNPs achieve LODs at the ng/mL level, and the spike recovery rates in fruit and vegetable samples are between 85.58% and 109.4%. Nevertheless, most existing UCNP-based systems are only applicable to the detection of single pesticide, and their capacity for simultaneous multi-component analysis needs further improvement [96]. Additionally, dual-mode techniques such as Raman-fluorescence [97,98] and fluorescence-colorimetry [99] have gradually gained attention. These hybrid techniques combine the advantages of two detection modalities to realize qualitative identification and accurate quantification simultaneously [100]. For instance, a dual-mode sensor based on CQDs and AuNPs enables rapid detection of malathion in cabbages. Combined with a smartphone, naked-eye semi-quantitative analysis can be accomplished, with sample recovery rates ranging from 88.7% to 107.6%, which is highly suitable for on-site screening [101].

Novel fluorescent probes represented by nitrogen-doped CQDs also show promising application prospects [102,103]. Fluorescence sensing systems established based on acetylcholinesterase inhibition can rapidly detect organophosphorus pesticides in fruit juices, with LODs of 2.89 ng, 4.69 ng and 6.40 ng for chlorpyrifos, trichlorfon and dufulin, respectively. The entire detection process takes less than 20 min, and the systems are characterized by low toxicity and strong anti-interference ability [104]. Another carbon dot-based colorimetric-fluorescent dual-mode system achieves an ultra-low LOD of 0.4 ng/mL for paraoxon with a wide linear range, providing a new approach for trace analysis of organophosphorus pesticides [105].

In short, diverse nanomaterials improve fluorescence-based pesticide detection: MOFs facilitate on-site testing, while signal amplification and ratiometric fluorescence resolve spectral overlap and poor stability via dual-modal design. Existing studies have demonstrated that nanomaterial-mediated fluorescence spectroscopy has become a vital development direction for pesticide residue detection, effectively overcoming multiple inherent limitations of traditional fluorescence detection. In the future, with the advancement of controllable preparation, surface modification, and multi-technology integration of nanomaterials, this research field will evolve toward higher sensitivity, faster response, better portability, and greater intelligence, providing more reliable technical support for food and environmental safety monitoring.

### 3.3. Other Spectral Detection Technologies

Apart from SERS and fluorescence spectroscopy, nanomaterials also play crucial roles in various other spectroscopic detection techniques, such as infrared spectroscopy and ultraviolet-visible (UV-Vis) absorption spectroscopy, with their functional mechanisms predominantly centered on optical property regulation. By modulating particle size, morphology, and microstructure, nanomaterials can optimize the light absorption and scattering behaviors of detection systems. Additionally, they serve as efficient signal amplification media and enrichment carriers to strengthen the spectral response intensity of target pesticides, thereby improving detection sensitivity and signal-to-noise ratio. Benefiting from excellent optical tunability and signal amplification capability, nanomaterials effectively compensate for the inherent deficiencies of conventional spectroscopic methods and significantly optimize the comprehensive detection performance of diverse spectral analytical systems. Such nanomaterial-enhanced spectroscopic techniques exhibit strong applicability in trace pollutant analysis and rapid screening scenarios. Notably, the research and practical application of nanomaterials in infrared spectroscopic pesticide detection are relatively mature among all optical spectroscopic branches.

Infrared spectroscopy exhibits prominent application potential in pesticide residue detection [106,107]. Near-infrared spectroscopy features a rapid response and is applicable for the rapid screening of pesticide components, while mid-infrared spectroscopy possesses high resolution and excellent selectivity. It can acquire molecular vibration information for the structural identification of pesticides [108,109]. Nevertheless, conventional infrared spectroscopy suffers from insufficient sensitivity and weak spectral signals of micro-residues, making it difficult to achieve accurate trace detection. Surface-enhanced infrared absorption spectroscopy (SEIRA) relies on metallic nanostructures to amplify spectral signals through electromagnetic and interfacial coupling effects. This technique significantly reduces the LOD, and possesses the advantages of rapidity, non-destructiveness, and simple pretreatment procedures, which renders it highly suitable for on-site pesticide detection [107,109].

**Figure 5:**
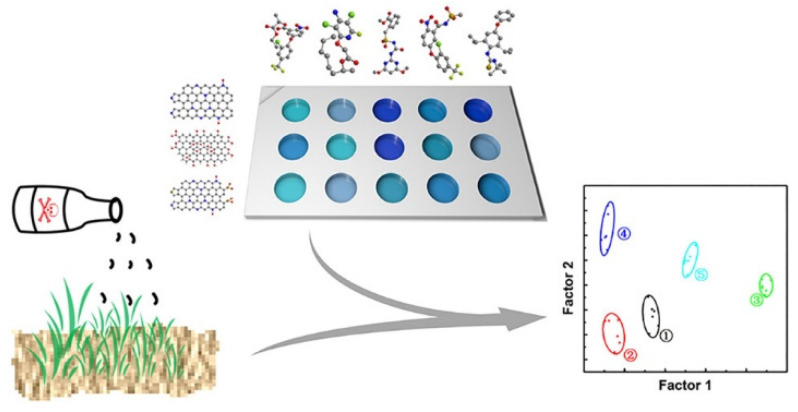
Nanozyme Sensor Arrays Based on Heteroatom-Doped Graphene for Pesticide Detection [110].

UV-Vis spectroscopy is also one of the mainstream techniques for the rapid detection of pesticide residues, and the introduction of nanomaterials has greatly optimized its comprehensive detection performance. For instance, AuNPs can enhance optical response signals relying on LSPR and catalytic effects, thereby substantially improving detection sensitivity. Nanozyme materials represented by heteroatom-doped graphene exhibit excellent peroxidase-mimicking activity. Such nanomaterials can reduce the detection cost by approximately 80%, improve the stability of detection systems, and realize the simultaneous identification of multi-component pesticides, showing remarkable practical application value. At present, UV-Vis spectroscopic detection mainly includes two mainstream strategies, namely derivatization and enzyme inhibition methods. The derivatization method can lower the LOD to 0.001 mg/kg, whereas it suffers from harsh reaction conditions and a relatively long detection period. By comparison, the enzyme inhibition method has favorable repeatability with a RSD lower than 3.0% and a rapid detection response that completes the entire test within several minutes, making it more suitable for on-site rapid screening (Figure 5) [110]. With the continuous advancement of portable detection technology, both strategies can be integrated with portable devices such as smartphones to further broaden practical application scenarios.

In conclusion, infrared and UV-Vis spectroscopy have been widely applied in pesticide residue detection. Their detection sensitivity, selectivity, and practicality have been substantially improved after modification with diverse nanomaterials. Infrared spectroscopy possesses unique advantages in molecular characteristic identification, making it suitable for rapid screening and structural analysis of pesticides. Nanoscale enhancement technologies such as SEIRA effectively address the weak signal limitation of conventional infrared spectroscopy, while innovative nano-substrates represented by CQDs further promote the accurate detection of trace pesticides. Relying on plasmonic effects and nanozyme-catalyzed reactions, UV-Vis spectroscopy has formed diversified detection modes including enzyme inhibition and derivatization, which can meet the differentiated requirements of on-site screening and laboratory trace analysis. Moreover, the development of nanozyme sensing arrays further enhances the anti-interference capability and simultaneous multi-pesticide detection performance in complex matrices. Nevertheless, both spectroscopic systems still suffer from inherent limitations, including poor reproducibility of nano-substrates, obvious environmental interference, and restricted application scope for target analytes. In the future, it is essential to optimize the fabrication processes of nanomaterials and combine multivariate modeling algorithms with portable device development to further improve spectroscopic detection systems. This optimization will provide robust technical support for the efficient, accurate, and on-site detection of pesticide residues.

### 3.4. Electrochemical Detection Technology

Nanomaterials with unique physicochemical properties play a pivotal role in the electrochemical and photoelectrochemical detection of pesticide residues. Their high specific surface area and excellent electrical conductivity can remarkably expand the active area of electrodes and accelerate interfacial electron transfer, thereby improving detection sensitivity and response rate. Through interfacial modification, host-guest recognition, and active site regulation, nanomaterials enhance the adsorption and selective recognition of target analytes and reduce background interference. Meanwhile, the construction of heterojunctions, core–shell structures, and spin-regulated systems can strengthen the separation and migration of photogenerated charge carriers, inhibit charge recombination, and achieve the amplification of photoelectric signals [111]. In addition, the synergistic effects among different nanomaterials enable interface optimization, signal amplification, and selective recognition, effectively overcoming the inherent drawbacks of traditional detection methods such as low sensitivity, poor stability, and narrow application range. These advantages provide critical technical support for the trace, rapid, and on-site detection of pesticides. For example, microelectrode arrays modified with β-cyclodextrin/multi-walled carbon nanotube composites can fully exploit the high conductivity and large specific surface area of carbon nanotubes. Combined with the host-guest inclusion effect of cyclodextrin, the composite improves analyte selectivity and realizes the rapid electrochemical detection of imidacloprid in vegetables. The sensing system exhibits a linear range of 5–100 μmol/L and a low LOD of 0.629 μmol/L, accompanied by favorable anti-interference capability, stability, and reproducibility. Moreover, its detection results are highly consistent with those obtained by HPLC-MS [112].

Electrochemical aptasensors have achieved groundbreaking advancements relying on nanomaterial-based interface engineering and signal amplification strategies [113,114]. For instance, Almenhali et al. constructed a hierarchical heterostructured electrochemical sensing platform based on Pt-doped MoS_2_ nanosheets decorated on Ti_3_C_2_ MXene. The fabricated composite integrates the high electrical conductivity of MXene, abundant active sites of MoS_2_, and superior electrocatalytic performance of Pt nanoparticles. Based on the enzyme inhibition mechanism, the sensor achieves the ultrasensitive detection of chlorpyrifos with a wide linear range from 10^−12^ to 10^−6^ M and an ultra-low LOD of 4.71 × 10^−13^ M. The spike recoveries in practical fruit and vegetable samples range from 94.81% to 104.0%, providing an efficient pathway for the trace detection of organophosphorus pesticides using two-dimensional nanomaterials [115]. Aiming to address the detection difficulty of acetamiprid with inherent electrochemical inactivity, Shi et al. fabricated a three-dimensional highly porous gold electrochemical impedance aptasensor. The porous nanostructure with large specific surface area and favorable biocompatibility efficiently immobilizes aptamers, significantly increasing the number of recognition sites and enhancing impedance signal intensity. The sensor presents a favorable linear response within 0.5–300 nmol/L, with a low LOD of 0.34 nmol/L. Its spike recoveries in fruit and vegetable samples are between 93.3% and 107.5%. This work provides a reliable strategy for the enzyme-free, highly sensitive, and on-site detection of neonicotinoid pesticides [116].

Defect-engineered graphene nanoribbons possess large specific surface areas, excellent electrical conductivity, and abundant defective active sites, which can effectively enhance the interfacial adsorption and electrocatalytic capability of electrodes. When applied for methyl parathion detection, the sensing system exhibits a linear range of 0.01–25.0 μM with an ultra-low LOD of 4.3 nM. The spike recoveries in actual water and fruit samples vary from 95.7% to 106.4%, providing a novel nanomaterial support for the high-efficiency sensing of pesticide residues [117]. The combination of nanomaterials with microfluidics and nanozyme technologies further realizes the integration of enrichment, catalysis, and detection. ZrO_2_@ZIF-90 bifunctional nanozymes integrate large specific surface area and phosphatase hydrolytic activity, which can efficiently enrich and catalytically degrade methyl parathion to generate electrochemically active products. This sensing system achieves an LOD of 0.53 μmol/L, with spike recoveries of 89.25–102.78% in practical samples. It offers an innovative insight for the synchronous degradation and trace detection of organophosphorus pesticides [118].

In the field of photoelectrochemical sensing, QDs, perovskites, and heterostructure designs provide feasible strategies for ultra-low limit-of-detection pesticide analysis. Self-powered photoelectrochemical aptasensors constructed based on Z-scheme perovskite heterostructures achieve the highly sensitive detection of profenofos in milk and cabbage through efficient interfacial carrier separation and suppressed charge recombination. This sensing system exhibits a linear range of 0.1–10^7^ ng/L with an ultra-low LOD of 0.033 ng/L, and the spike recoveries of practical samples are between 96.68% and 107.2% [119]. Wang et al. further fabricated a spin-reconfigurable magnetic perovskite paper-based sensing platform. CdS-coated perovskite QDs were synthesized to improve the aquatic environmental stability, while type-II heterojunctions were constructed by integrating AuNPs with Br, N co-doped TiO_2_. The external magnetic field significantly prolongs the carrier lifetime, enabling the ultra-trace detection of acetamiprid. The sensor achieves a wide linear range from 10^−13.5^ to 10^−9^ M and an extremely low LOD of 23 fm. The spike recoveries of actual fruit and vegetable samples are 90.7–105.6%, providing an innovative method for the on-site and highly stable detection of neonicotinoid pesticides [120].

In summary, benefiting from large specific surface areas, excellent electrical conductivity, and versatile interfacial regulation capabilities, nanomaterials perform critical functions in signal amplification and selectivity enhancement for electrochemical and photoelectrochemical pesticide detection. Composite nanostructures such as β-CD/MWCNT, Pt/MoS_2_/MXene, and porous gold can remarkably improve electron transport efficiency and target recognition ability, realizing the rapid and highly sensitive detection of imidacloprid, chlorpyrifos, acetamiprid, and other pesticides. Defect-engineered graphene nanoribbons and ZrO_2_@ZIF-90 nanozymes further enhance catalytic and enrichment performances, satisfying the requirements for efficient detection and degradation of organophosphorus pesticides. Emerging nanomaterials including perovskite heterojunctions and core–shell QDs optimize the separation and stability of photogenerated charge carriers, achieving ultra-trace pesticide detection. Various nanomaterials synergistically construct diversified sensing systems, providing solid technical support for the on-site, rapid, and precise detection of pesticide residues.

## 4. Comparison of Various Detection Methods

Table 2 summarizes a variety of pesticide residue detection technologies, mainly including SERS, electrochemical and fluorescence sensing platforms. This table also sorts out the nanomaterials adopted, analytical indicators and applicable pesticide categories of each method. A large number of functional nanomaterials have been applied in these sensing systems, such as gold and AgNPs, carbon-based materials, QDs, MXene, MOFs and core–shell nanocomposites. These nanomaterials are key to equipping different detection techniques with superior sensing sensitivity. 

Nanomaterial-based sensing technologies enable ultra-trace and accurate detection of pesticide residues with excellent LODs. The quantitative detection units of these methods cover mg/L, μg/kg and ng/mL, and even reach ultra-trace concentration rang-es of fg/mL, nmol/L and pmol/L. To unify the quantitative standard, the LODs of typical detection methods are converted into a unified unit of μg/kg. The integration of func-tional nanomaterials and innovative sensing strategies significantly improves the ul-tra-trace analytical performance for various agrochemicals, including organophospho-rus pesticides, neonicotinoid insecticides, herbicides and fungicides, achieving a sub-stantial enhancement in detection sensitivity. Specifically, the aptamer-based SERS bi-osensor exhibits an LOD of 0.00147 ng/mL (0.00147 μg/kg) for the herbicide 2,4-D [70], while the palladium nanoparticle and cadmium sulfide-modified photoelectrochemical sensor achieves an ultra-low LOD of 3.3 × 10^−13^ mol/L (6.30927 × 10^−5^ μg/kg) for the fun-gicide carbendazim [114]. These results fully demonstrate the ultra-low detection level and superior ultra-trace detection capability of such nanosensing technologies. With regard to detection precision and repeatability, most existing techniques yield acceptable recoveries between 80% and 120% when applied to various food and agricultural samples. For example, SERS combined with deep learning gains recovery values of 90–115% in the detection of chlorpyrifos [52], while fluorescent probes obtain 95–99% recoveries for methyl parathion [51]. The RSD of most tests is controlled below 10%, and several methods even deliver RSD less than 3%. The ratiometric fluorescent probe for methyl parathion [51] and the fluorescence-magnetic separation method for thiram [88] are typical representatives, which fully verify the excellent reproducibility and stability of these analytical approaches.

Noticeable differences in testing efficiency exist among different detection routes. Most SERS, electrochemical and traditional fluorescence methods can finish the whole detection process within several minutes to half an hour, which perfectly meets the requirement of rapid field screening. By contrast, dual-channel fluorescence detection and molecular imprinting-based fluorescence assays generally spend 18 to 60 min on analysis [69,101,121]. A small number of technologies like SERS-aptamer sensing and nanozyme sensors also need relatively longer testing time [70,118]. In addition, the introduction of MIPs, aptamers, machine learning and chemometric algorithms can further enhance the anti-interference capability, target specificity and comprehensive detection performance.

Overall, multi-modal analytical techniques constructed from functional nanomaterials show distinctive strengths in pesticide residue monitoring. Benefiting from diversified nanomaterials, novel recognition elements and intelligent data processing technologies, these approaches can satisfy the practical requirements of high sensitivity, reliable accuracy and fast detection for different types of pesticides. As reliable analytical tools, they possess promising application potential in food safety control and agricultural product quality testing.

## 5. Problems and Challenges of Nanomaterials in Pesticide Detection Applications

### 5.1. Inherent Deficiencies of Nanomaterials

The inherent drawbacks of nanomaterials severely restrict their large-scale application in pesticide detection. Firstly, poor batch-to-batch homogeneity remains a critical issue. Variations in particle size, morphology, and surface physicochemical properties across different batches inevitably cause unstable sensing signals and inferior detection repeatability. Secondly, certain nanomaterials exhibit unsatisfactory biocompatibility. For instance, QDs and noble metal nanoparticles possess potential biological toxicity, limiting their practical applicability in food and aquatic matrices that are closely related to human health. Thirdly, immature surface modification techniques commonly induce nanoparticle aggregation, which reduces specific surface areas and diminishes active sensing sites. This phenomenon further weakens the signal amplification capability of nanosensors. Additionally, complicated modification procedures and high raw material costs hinder scalable industrial fabrication. Moreover, residual modifiers may introduce non-specific interference, thereby lowering detection accuracy.

### 5.2. Technical Challenges of Current Detection Systems

Current nanomaterial-based detection technologies still face multiple technical bottlenecks in precise pesticide quantification. On the one hand, insufficient compatibility between nanomaterials and analytical techniques frequently induces signal quenching and uncontrollable background interference [121]. The synergistic modulation between nano-substrates and spectroscopic/electrochemical platforms requires further optimization. On the other hand, most existing sensors are only capable of detecting a single or a small number of pesticide components, which cannot satisfy the practical demand for simultaneous multi-residue analysis in complex real matrices [122]. Furthermore, sensing signals are highly susceptible to external environmental fluctuations, including temperature and pH variations. Such environmental sensitivity inevitably generates quantitative deviations, making it difficult to meet standardized detection requirements [123].

### 5.3. Practical Application Restrictions

Several practical obstacles impede the commercial promotion and on-site implementation of nanomaterial-based sensing techniques. Primarily, the anti-interference ability in complex matrices remains insufficient. Complex endogenous components in agricultural products, water, and soil (e.g., proteins, lipids, and humic substances) tend to bind with nanomaterials, causing signal distortion, elevated detection limits, and deteriorated quantification accuracy. Secondly, most high-performance nanosensors rely on bulky laboratory instruments and tedious operation procedures, accompanied by prolonged detection durations, which severely limits their feasibility for rapid field screening. Thirdly, the high synthesis cost of premium nanomaterials restricts industrialization. Moreover, the absence of unified testing criteria and standardized operation protocols leads to poor result reproducibility among different laboratories, further hindering the large-scale commercialization of nanosensing platforms.

## 6. Future Trends and Perspectives

### 6.1. Development Trends of Nanomaterials in Agriculture

Green chemistry and cost-effective fabrication will become the primary developmental directions for agricultural nanomaterials. Conventional synthetic routes generally rely on toxic chemical reagents and cumbersome fabrication processes. In the future, considerable efforts will be devoted to exploring eco-friendly synthetic strategies, where biomass and natural extracts are adopted as low-cost and sustainable precursors to fabricate environmentally benign nanomaterials. Such green fabrication protocols can effectively reduce manufacturing costs and mitigate potential ecological risks. Additionally, precise functional modification is regarded as a core developmental tendency. Rational surface engineering can optimize the structural stability, molecular specificity, and biocompatibility of nanosystems, thereby ameliorating the inherent defects including poor batch uniformity and irreversible aggregation. Moreover, the functional evolution from single-target identification to multi-functional integration is anticipated to realize the adaptive application of nanomaterials in pesticide monitoring, soil remediation, and crop nutrient regulation.

### 6.2. Future Prospects

Nanomaterials are expected to be deeply integrated into the entire agricultural industrial chain to address the core bottlenecks restricting modern agricultural development. In terms of pesticide detection, miniaturized and portable nanosensing devices will be continuously developed to achieve rapid on-site screening and simultaneous multi-residue quantification. The establishment of unified and standardized detection systems will further promote the scalable industrialization of nanosensors. With respect to agricultural production and environmental governance, engineered nanomaterials can serve as efficient carriers for the controlled release of pesticides and fertilizers, which effectively alleviates agricultural non-point source pollution. Furthermore, nanomaterial-based remediation strategies exhibit promising potential for the elimination of heavy metals and residual pesticides in contaminated soil, contributing to the optimization of agricultural ecological environments. In addition, in-depth systematic research is required to resolve the inherent limitations concerning biological safety and long-term structural stability. The in-depth integration of nanotechnology and modern agriculture will provide robust technical support for guaranteeing agricultural product safety and realizing green and sustainable agricultural development.

## 7. Conclusions

This review systematically summarizes the classification and application performance of diverse nanomaterials in pesticide residue detection, including metallic nanomaterials, metal oxides, carbon-based materials, MOFs, and QDs. The intrinsic superiorities of nano-sensors in high sensitivity, favorable selectivity, and rapid analytical efficiency are highlighted, and the latest research advances in spectroscopic and electrochemical detection systems are comprehensively reviewed. Meanwhile, the current technical and application bottlenecks are explicitly clarified, covering insufficient batch homogeneity, unsatisfactory biosafety, weak anti-interference capability in complex matrices, and the lack of universal industrial standards. In the future, nanomaterial-based sensing techniques will evolve toward green synthesis, precise functionalization, intelligent portability, and multi-component synchronous detection. With the continuous breakthroughs in fabrication engineering, interfacial modification, and multi-technology integration, nano-sensing platforms will achieve large-scale industrial implementation. Collectively, this review demonstrates the profound application value of nanotechnology, which is of great significance for food safety guarantee, ecological environmental protection, and the sustainable development of modern green agriculture.

## Figures and Tables

**Table 1:** Categories of Pesticides Investigated in This Study.

Pesticide Use Category	Pesticide Chemical Class	Pesticide Common Name	Chemical Structural Formula
Insecticide	Organophosphorus	Chlorpyrifos	O,O-Diethyl O-(3,5,6-trichloro-2-pyridinyl) phosphorothioate
Insecticide	Organophosphorus	Malathion	Diethyl (dimethoxyphosphinothioylthio)succinate
Insecticide	Organophosphorus	Parathion-methyl	O,O-Dimethyl O-(4-nitrophenyl) phosphorothioate
Insecticide	Organophosphorus	Paraoxon-ethyl	O,O-Diethyl O-(4-nitrophenyl) phosphate
Insecticide	Organophosphorus	Profenofos	O-Ethyl O-4-bromo-2-chlorophenyl S-propyl phosphorothioate
Insecticide	Neonicotinoid	Acetamiprid	(E)-N-[(6-Chloro-3-pyridyl)methyl]-N’-cyano-N-methylacetamidine
Insecticide	Neonicotinoid	Imidacloprid	1-(6-Chloro-3-pyridylmethyl)-N-nitroimidazolidin-2-ylideneamine
Insecticide	Pyrethroid	Deltamethrin	(S)-α-Cyano-3-phenoxybenzyl (1R,3R)-3-(2,2-dibromovinyl)-2,2-dimethylcyclopropanecarboxylate
Insecticide	Pyrethroid	Cyfluthrin	(RS)-α-Cyano-4-fluoro-3-phenoxybenzyl (1RS,3RS;1RS,3SR)-3-(2,2-dichlorovinyl)-2,2-dimethylcyclopropanecarboxylate
Insecticide	Others	Fipronil	(RS)-5-Amino-1-(2,6-dichloro-4-trifluoromethylphenyl)-4-trifluoromethylsulfinylpyrazole-3-carbonitrile
Insecticide	Others	Nereistoxin Insecticides	(1,2-Dithiolan-4-yl) dimethylamine
Fungicide	Benzimidazole	Carbendazim	Methyl 2-benzimidazolecarbamate
Fungicide	Benzimidazole	Thiabendazole	2-Thiazol-4-ylbenzimidazole
Fungicide	Dithiocarbamate	Thiram	Tetramethylthiuram disulfide
Fungicide	Organochlorine	Quintozene	1,2,3,4,5-Pentachloro-6-nitrobenzene
Fungicide	Others	Chloramphenicol	2,2-Dichloro-N-[(1R,2R)-1,3-dihydroxy-1-(4-nitrophenyl)propan-2-yl]acetamide
Herbicide	Phenoxycarboxylic Acids	2,4-Dichlorophenoxyacetic Acid (2,4-D)	2,4-Dichlorophenoxyacetic acid
Insecticide	Organophosphorus	Chlorpyrifos	O,O-Diethyl O-(3,5,6-trichloro-2-pyridinyl) phosphorothioate

**Table 2:** Detection performance comparison of various detection methods.

Detection Scheme	Data Analysis Method	Nanomaterials Used	Detection Matrix	Pesticides	Pesticide Categories	Recovery Rate (%)	RSD (%)	LOD (μg/kg)	Detection Time	Reference
Aptamer	Fluorescence/Colorimetric	AuNPs; GO; Magnetic nanoparticles (MNPs)	Milk; Food	Chlorpyrifos; Methamidophos; Dimethoate	Organophosphorus P (OPPs)	94.4–102.8%	-	Chlorpyrifos: 59.60∼8414.16; Methamidophos: 23.99∼3386.88; Dimethoate: 38.97∼5502.24;	Rapid	[20]
SERS-Aptamer	SERS	AgNPs; Silver anostars; AuNPs	Wheat samples	Omethoate; Dimethoate; Isocarbphos; Monocrotophos	OPPs	97.25–119.38%	-	0.558	Minute-level	[22]
MIPs-SERS	SERS	AgNPs; MIPs	Milk	2,4-D	Herbicide (Phenoxycarboxylic Acid Class)	85–95%	<10%	6	20 min	[25]
MIPs-SERS	SERS	AuNPs; MIPs	Wheat/Rice	Prometryn, Simetryn	Herbicide (Triazine Class)	72.7–90.9%	1.7–7.8%	20	Usually < 30 min)	[28]
SERS Solid Substrate	SERS	Ag/Au nanodendrites	Apple juice	Thiram	Fungicide	94.8–97.2%	3.53–4.49%	86.1	-	[34]
SERS - ptamer	SERS	Unmodified AuNPs	Tea	Acetamiprid	Neonicotinoid Insecticide	98.45–104.5%	<5%	3.919	Rapid	[36]
Colorimetric Method	UV-Vis Spectroscopy	AuNPs	Tea; Kiwi fruit	Bisultap; Monosultap; Cartap	Insecticide	90–102%	5.0–10.9%	40 (Instrumental); 50–100 (Visual)	3 min	[38]
Electrochemical Biosensor	Electrochemical Signal	Ti_3_C_2_Tx MXene	fruits and vegetables; Paddy water	Carbendazim	Fungicide	-	-	0.19119	Rapid	[39]
SERS -Machine Learning	SERS	Ag@ZnO NFs	Wheat	Deltamethrin	Insecticide (Pyrethroid Class)	96.33–109.17%	<5%	0.16	Minute-level	[40]
Photoelectrochemical/Immunoassay	Photocurrent/Fluorescence	g-C_3_N_4_; CdTe QDs	Milk; Agricultural products	Carbofuran	Carbamate Insecticide	92.18–110.72%	2.88–15.97%	0.4447	Rapid (DPV detection)	[48]
Ratiometric Fluorescent Probe	Fluorescence	SiQDs	Fruit and vegetable food products	Methyl Parathion	Organophosphorus Insecticide	95–99%	<3.0%	0.0392	30–60 min	[51]
SERS + Deep Learning	SERS	Au@Ag core–shell nanoparticles (CS NPs)	Fruit and vegetable	Chlorpyrifos	Organophosphorus Insecticide	90–115%	<10% (Most < 5%)	0.00011∼0.0157	<20 min	[52]
SERS - Chemometrics	SERS	AgNFs	Rice	Chlorpyrifos; Carbendazim	Organophosphorus InsecticideBenzimidazole Fungicide	No significant difference compared with HPLC	<5.55%	10	Rapid	[63]
Fluorescence/Photothermal Multimode	Fluorescence/Photothermal Spectroscopy	r-CDs@CoOOH NSs	Leafy vegetables; tangerine; orange	Fenitrothion	Organophosphorus Insecticide	Consistent with GC-MS/MS	<18.5%	0.14	55 min	[69]
SERS Aptamer Sensor	SERS	AuNPs; magnetic MIP	Milk; Water samples	2,4-D	Herbicide (Phenoxycarboxylic Acid Class)	93.5–102.2%	<5%	0.00147	<2 h	[70]
Fluorescence/Enzyme Inhibition	Fluorescence Spectroscopy	CdTe QDs; AChE aerogel	Fruit samples	Paraoxon; Parathion,	Organophosphorus Insecticides	98–110%	<10%	Paraoxon: 0.000105∼0.00033; Parathion: 0.000111∼0.00035	<20 min	[85]
Fluorescence/Magnetic Separation	Fluorescence Spectroscopy	UCNPs; Dithizone-Cd^2+^ Composite	Apple	Thiram	Fungicide (Dithiocarbamate Class)	90.53–112.39% (vs. HPLC)	0.01–2.88%	6.75	Rapid	[88]
Fluorescence/Enzyme Inhibition	Fluorescence Spectroscopy	AuNPs	Rice; Fruit and vegetable foods	Chlorpyrifos	Organophosphorus Insecticide	-	-	0.7012	Not explicitly mentioned	[90]
Fluorescence/Colorimetric Dual-Mode Sensing	Fluorescence/UV-Vis Spectroscopy	CQDs; GNPs	Chinese cabbage	Malathion	Organophosphorus Insecticide	89.9–103.4% (Fluorescence method); 88.7–107.6% (Colorimetric method)	-	0.0429 (Fluorescence method); 0.1949 (Colorimetric method)	30 min	[101]
Fluorescence/Enzyme Inhibition	Fluorescence Spectroscopy	CdTe QDs	Rice; Banana; Potato; Garlic	Methyl Parathion	Organophosphorus Insecticide	90–105%	-	15.7926	-	[102]
Fluorescence/Enzyme Inhibition	Fluorescence Spectroscopy	N-CQDs	Apple juice; Orange juice	Chlorpyrifos; Trichlorfon; Dufulin	Organophosphorus Insecticide; Fungicide	85.33–110.67%	4.06–9.27%	2.89–6.40	<20 min	[104]
Dual-Mode Sensing	Fluorescence Spectroscopy	CDs	Tap water; Rice; Cabbage	Paraoxon	Organophosphorus Insecticide	90–102%	<4.17%	0.4	-	[105]
Electrochemical Enzyme Sensor	Differential Pulse Voltammetry (DPV)	β-CD/MWCNT	Cabbage; Cucumber; Tomato	Imidacloprid	Neonicotinoid Insecticide	94.5–112.92% (vs. HPLC-MS)	<5.4%	160.83	Rapid	[112]
Photoelectrochemical Aptamer Sensor	Photoelectrochemical (PEC) Signal	Pd NPs/CdS	Lettuce	Carbendazim	Benzimidazole Fungicide	98.93–106.10%		6.30927 × 10^−5^	Usually < 30 min	[114]
Electrochemical Enzyme Sensor	DPV	Pt-dopedMoS_2_/Ti_3_C_2_ MXene (Pt/MoS_2_/TM)	Fruits, vegetables and their washing water	Chlorpyrifos	Organophosphorus Insecticide	94.81–104.0%	2.6–5.3%	1.6512789 × 10^−4^	20 min (Inhibition time)	[115]
Electrochemical Aptamer Sensor	Electrochemical Impedance Spectroscopy (EIS)	Porous gold nanostructures	Apple; Pear; Orange; Cucumber; Tomato; Pakchoi	Acetamiprid	Neonicotinoid Insecticide	93.3–107.5%	4.16%	0.0757	40 min	[116]
Electrochemical Enzyme Sensor	DPV	Defect-engineered graphene nanoribbons	Tap water; Lake water; Tomato juice	Methyl Parathion	Organophosphorus Insecticide	95.7–106.4%	-	1.1318	Rapid	[117]
Nanozyme/Bifunctional Sensor	DPV	ZrO_2_@ZIF-90	Apple; Pear	Methyl Parathion	Organophosphorus Insecticide	89.25–102.78%	2.35–7.06%	139.5013	Long	[118]
Photoelectrochemical Aptamer Sensor	Photocurrent	Perovskite heterojunction	Milk; Cabbage	Profenofos	Organophosphorus Insecticide	96.68–107.2%	1.08–4.54%	0.000033	Rapid	[119]
Fluorescence/Molecular Imprinting	Fluorescence	CdTe QDs/SiO_2_	Sprout juice; Bean sprout	2,4-D	Herbicide	94–107%	-	0.4642	18 min	[121]
Fluorescence/Molecular Imprinting	Fluorescence	CdSe/ZnS QDs	Cucumber; Eggplant	Parathion-methyl	Organophosphorus Insecticide	-	-	4	20 min	[121]
Fluorescence/Molecular Imprinting	Fluorescence	SiCQDs	Well water; Apple; Tomato	Indoxacarb	Insecticide	95–106%	-	0.528	5 min	[121]
SERS/Aptamer	SERS	Au/Ag NPs	Citrus; Tea; Tomato	Chlorpyrifos	Organophosphorus Insecticide	-	-	0.35059 × 10^−3^	Rapid	[122]
SERS/Molecular Imprinting	SERS	ZnO/GO/Ag composite	Songhua River water	Cyfluthrin	Pyrethroid Insecticide	97.34–104.23%	-	0.017372	Usually < 30 min	[123]

Note: All LOD values were unified as μg/kg.

## Data Availability

No new data were created or analyzed in this study. Data sharing is not applicable to this article.
